# Zero‐echo‐time sequences in highly inhomogeneous fields

**DOI:** 10.1002/mrm.30352

**Published:** 2024-10-21

**Authors:** Jose Borreguero, Fernando Galve, José M. Algarín, Joseba Alonso

**Affiliations:** ^1^ MRILab, Institute for Molecular Imaging and Instrumentation (i3M), Spanish National Research Council (CSIC) Universitat Politècnica de València (UPV) Valencia Spain; ^2^ Tesoro Imaging S.L. Valencia Spain

**Keywords:** artifact correction, field inhomogeneity, low field, ultra‐short $T_{2}^{*}$, zero echo time

## Abstract

**Purpose:**

Zero‐echo‐time (ZTE) sequences have proven a powerful tool for MRI of ultrashort $T_{2}$ tissues, but they fail to produce useful images in the presence of strong field inhomogeneities (14 000 ppm). Here we seek a method to correct reconstruction artifacts from non‐Cartesian acquisitions in highly inhomogeneous $B_{0}$, where the standard double‐shot gradient‐echo approach to field mapping fails.

**Methods:**

We present a technique based on magnetic field maps obtained from two geometric distortion‐free point‐wise (SPRITE) acquisitions. To this end, we employ three scanners with varying field homogeneities. These maps are used for model‐based image reconstruction with iterative algebraic techniques (ART). For comparison, the same prior information is fed also to widely used Conjugate Phase (CP) algorithms.

**Results:**

Distortions and artifacts coming from severe $B_{0}$ inhomogeneities, at the level of the encoding gradient, are largely reverted by our method, as opposed to CP reconstructions. This holds even close to the limit where intra‐voxel bandwidths (determined by $B_{0}$ inhomogeneities, up to 1.2 kHz) are comparable to the encoding inter‐voxel bandwidth (determined by the gradient fields, 625 Hz in this work).

**Conclusion:**

We have benchmarked the performance of a new method for ZTE imaging in highly inhomogeneous magnetic fields. For example, this can be exploited for dental imaging in affordable low‐field MRI systems, and can be expanded for arbitrary pulse sequences and extreme magnet geometries, as in, for example, single‐sided MRI.

## 1 INTRODUCTION

Producing diagnostically valuable images of biological tissues with ultra‐short (sub‐ms) $T_{2}$ is a long standing goal in MRI research.^1^ Special‐purpose pulse sequences such as Ultra‐short Echo Time, ZTE (Zero Echo Time) and Sweep Imaging with Fourier Transformation have been crucial, and can be employed for a variety of applications, including dental, lung, musculoskeletal, or myelin imaging.^2^, ^3^, ^4^, ^5^ Among these sequences, ZTE imposes the most stringent constraints on hardware specifications, but it is best suited for MRI of tissues with sub‐ms $T_{2}$.^6^

The advent of clinically viable low‐field MRI (LF‐MRI) technologies has brought along a plethora of new possibilities which can be realized by translating clinical high‐field techniques to the low‐field regime, at a lower cost and in a more accessible fashion.^7^, ^8^, ^9^ Imaging of ultra‐short $T_{2}$ tissues, however, is not one of them. While in vivo dental human MRI has been demonstrated at $B_{0}\geq 3$ T,^10^, ^11^ it remains a pending challenge in the sub‐tesla regime, mostly due to lack of signal‐to‐noise ratio (SNR). ZTE‐like sequences such as PETRA (Pointwise Encoding Time reduction with Radial Acquisition^12^) have been used for simultaneous soft and hard tissue imaging at low fields (260 mT), but only with ex vivo samples.^13^ Furthermore, these systems feature small FOVs and are incompatible with in vivo clinical operation.^14^, ^15^ To explore the viability of hard‐tissue in vivo LF‐MRI, we built a custom 197 mT yoked magnet for which, due to geometric and weight constraints, the principal magnetic field is extremely inhomogeneous and standard reconstructions suffer from severe artifacts.

In this paper, we first describe a method to measure field inhomogeneity in a reliable way, and show that the usual double‐shot gradient‐echo (GRE) technique^16^ fails beyond usability due to irrecoverable intra‐voxel phase accumulation and geometric misassignment of spatial coordinates. Our method, although more time‐costly in certain contexts, can cope with extreme field inhomogeneities, leveraging a double‐shot scheme with single‐point acquisitions which accumulate a single, different, phase per shot (Single‐Point Double‐Shot, SPDS). Using the acquired image‐based $B_{0}$ map as prior knowledge (PK), we build an encoding matrix (EM) for algebraic reconstruction through Kaczmarz's algorithm (ART^17^), which yields faithful images in situations where Conjugate‐Phase reconstruction^18^ fails. The combination of SPDS and ART works even near the limit where the applied encoding gradient spans an inter‐voxel bandwidth (BW) similar to the intra‐voxel bandwidth induced by off‐resonance ($\Delta B_{0}$). We demonstrate the benefits of the method for linear and quadratic inhomogeneities, as well as for fields with finer spatial details and abrupt variations.

## 2 METHODS

### 2.1 MRI scanners and samples

**FIGURE 1:**
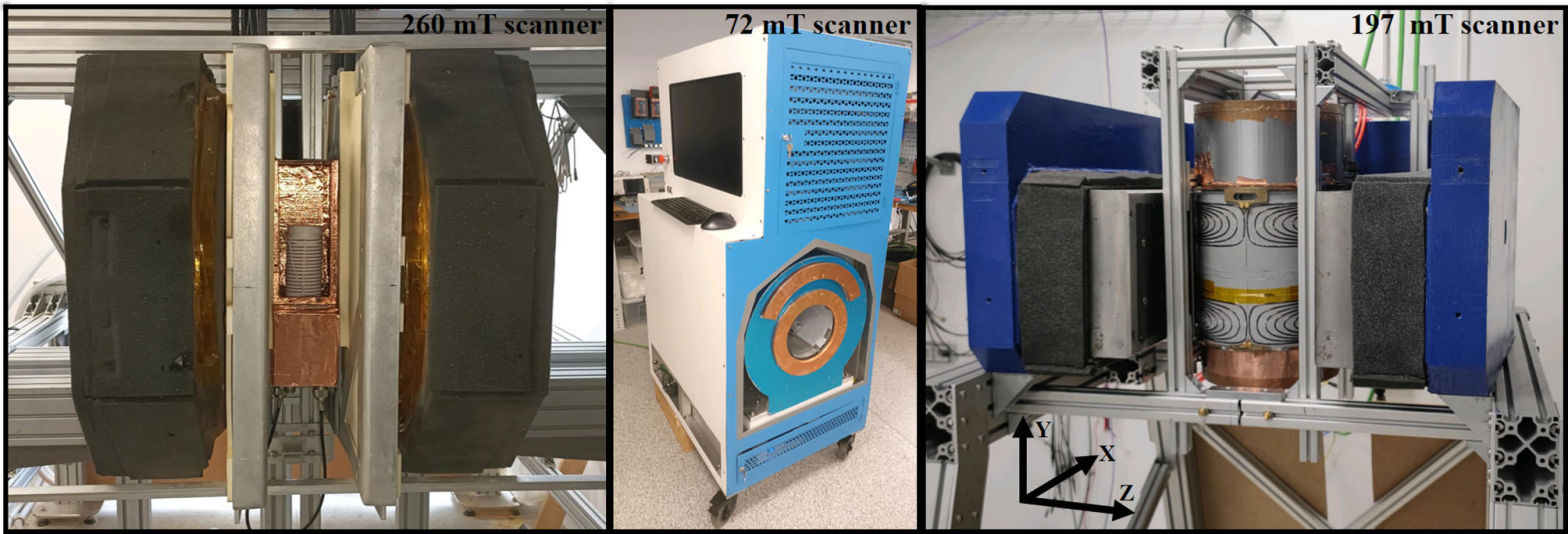
The three scanners used for our experiments. (Left) 260 mT scanner with 20 ppm inhomogeneity in a 150‐mm diameter field of view (FOV). (Middle) 72 mT scanner with 3,100 ppm in a 200‐mm diameter FOV. (Right) 197 mT scanner with 14 000 ppm in a 92×71×110 mm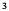 FOV.

We have carried out experiments in the three MRI scanners shown in Figure 1: (i) a highly homogeneous 260 mT C‐shaped yoked magnet (described previously^13^); (ii) a reasonably homogeneous 72 mT Halbach array‐based scanner (described previously^9^); and (iii) a recently built, highly inhomogeneous 197 mT scanner purpose‐designed for dental in vivo imaging.

**FIGURE 2:**
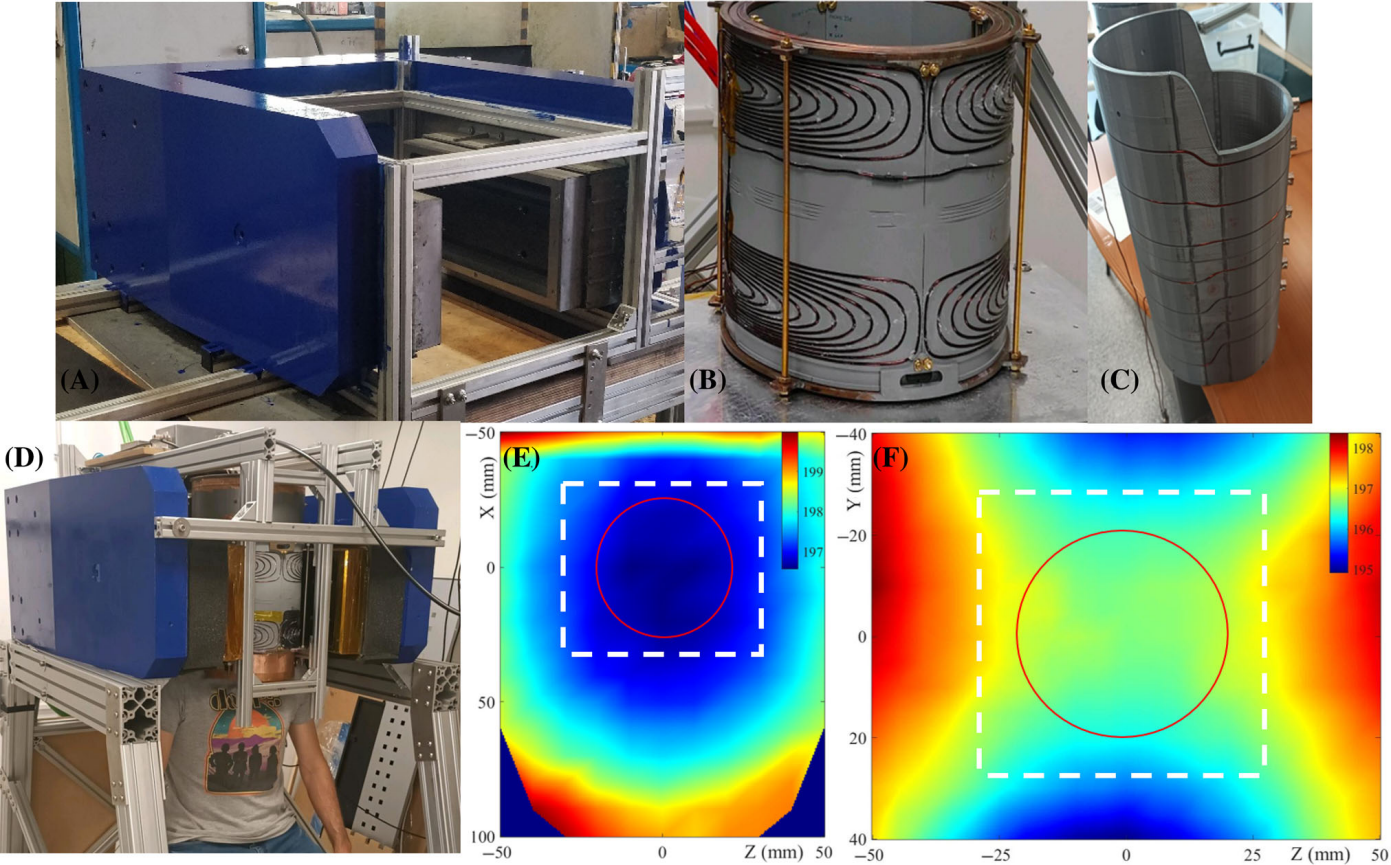
197 mT scanner designed for low‐field dental MRI. (A) Yoked magnet with shimming units. (B) Water‐cooled gradient system. (C) Radiofrequency excitation coil. (D) Fully assembled scanner. (E) Principal magnetic field $B_{0}$ in the xz plane (y=0). (F) Field in the yz plane (x=0). The square represents the (6 cm)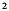 field of view, while the circle represents the 4‐cm diameter i3M phantom.

The latter scanner (see Figure 2) has a C‐shaped yoked magnet providing a 197 mT main field, where the gap between poles is 27 cm and the total weight is around 1200 kg. The field inhomogeneity has been measured to be around 14 000 ppm (parts per million) in a FOV of 92×71×110 mm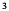, and the spatial distribution is mostly quadrupolar at its center. The encoding gradient coils are formed by wires wound around water‐cooled copper plates, and can achieve a maximum of ≈76,27,51 mT/m strengths (at 100 A) along the x, y, z directions, respectively. The transmission (Tx) coil of the radiofrequency (RF) system is a solenoid with Q≈52 and can produce a π/2 pulse in ≈ 16 μs. This provides a mostly uniform excitation profile for 75 kHz bandwidths (1.8 mT), covering even the largest inhomogeneities studied in this work (21 kHz in Figures 7B,E). We employ a variety of reception (Rx) coils, which we typically adjust for maximum SNR with different samples.

All phantoms used for reconstruction studies are replicas of a polylactic acid three‐dimensional (3D)‐printed hollow cylinder with the characters “i3M” inside, with 4 cm outer diameter and character thickness 2–3 mm. The exception is a 8×13 cm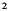 rectangular phantom with 6–7 mm thick characters in Figure 7F, due to the larger available FOV in the 72 mT scanner. For $B_{0}$‐mapping studies (Figure 5) we use an empty hexagonal phantom with 4 cm outer diameter. Although we employ ZTE sequences, specifically PETRA, because we seek compatibility with ultra‐short $T_{2}$ imaging, we fill the phantoms with a 1% CuSO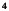 water solution. This features $T_{2}\approx 7.5$ ms (measured with CPMG^19^, ^20^) and $T_{1}\approx 16$ ms (Inversion‐Recovery,^21^) in the 260 mT scanner. At any rate, for the ZTE sequences used in this article $T_{2}^{*}$ is most relevant and is as low as 750 μs for the cases considered here.

### 2.2 Field estimation

We estimate $B_{0}$ with three different techniques: (i) a Hall probe scanning the FOV volume guided by a robot; (ii) the usual double‐shot echo‐time‐shifted GRE^16^; and (iii) our double‐shot single‐point Cartesian acquisition (SPDS), where each shot provides a different encoding time.

#### 2.2.1 Hall sensor

For the first procedure we use a THM1176^22^ magnetometer mounted on a home‐made xyz linear stage to measure $B_{0}(\vec{x})$ on a Cartesian grid over a volume 15×10×8 cm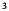 with isotropic spacing of 1 cm. We strived to reference the $B_{0}$ map to image space by finding the saddle point of the measured field and matching it to the image.

#### 2.2.2 Double‐shot GRE

The double‐shot GRE method uses two low‐resolution images from two GRE sequences with one echo per repetition, where the echo time (TE) is different for each acquisition.^16^ Typically, the k‐spaces are Fourier‐transformed to produce images $\rho_{1}$ and $\rho_{2}$. Since most of the signal energy is concentrated on the echo, one usually assumes that the phases (arg) of the images differ by $\approx \gamma \Delta B_{0}({\text{TE}}_{2}-{\text{TE}}_{1})$ in all voxels, where γ is the g‐factor and $\Delta B_{0}$ is the off‐resonance at each voxel. The field can then be estimated as:

(1) $$\Delta B_{0}\approx \frac{\arg(\rho_{2})-\arg(\rho_{1})}{\gamma \left({\text{TE}}_{\text{2}}-{\text{TE}}_{\text{1}}\right)}.$$

GRE sequences are inherently sensitive to field inhomogeneity. To minimize geometric distortions, it is convenient to fix TE and the readout window duration as short as possible. However, this is ultimately limited by hardware, hence images invariably suffer from nonnegligible geometric distortions in our regime of extreme off‐resonance.

#### 2.2.3 SPDS

**FIGURE 3:**
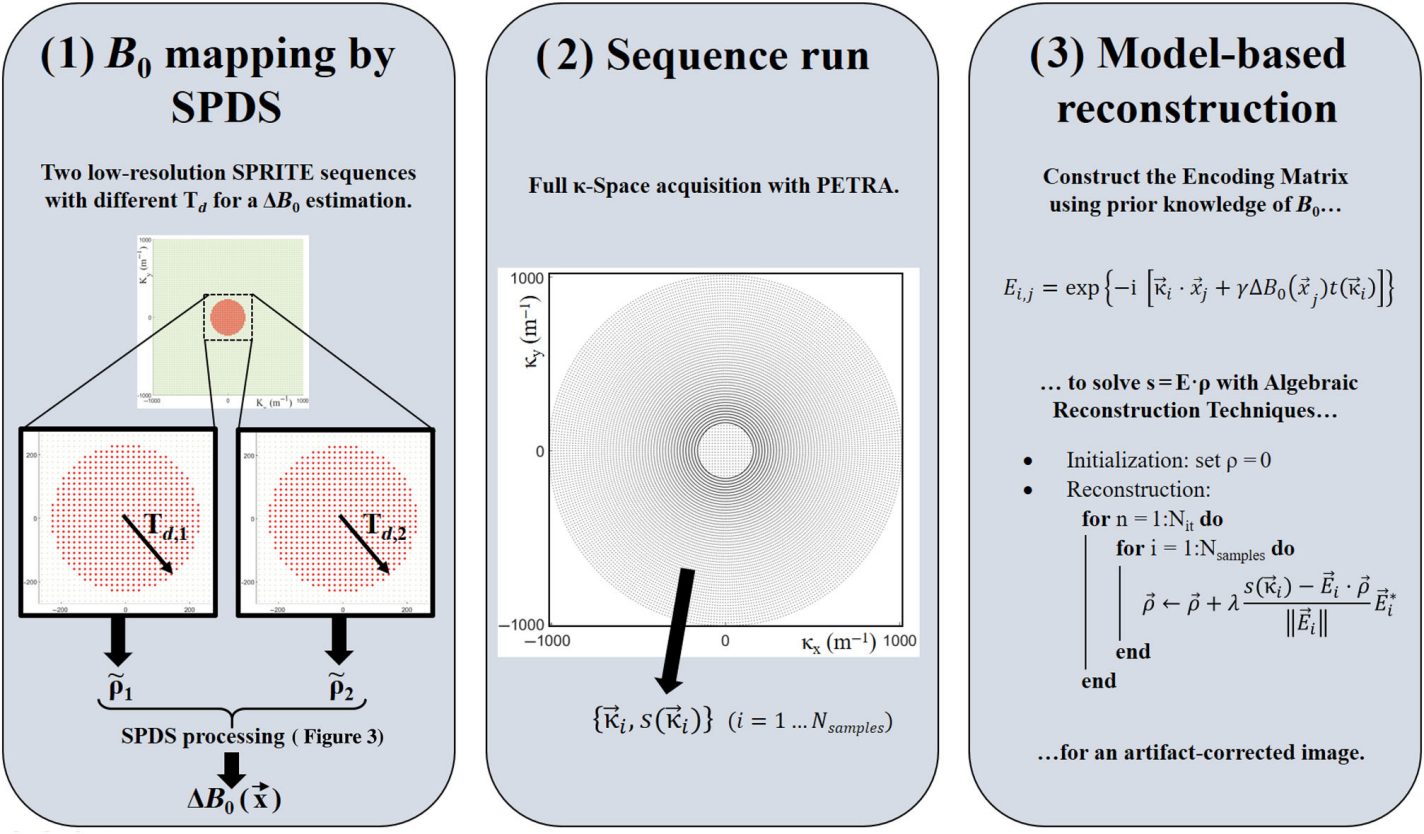
Diagram of the proposed procedure for zero‐echo‐time (ZTE) imaging in highly inhomogeneous fields. (1) Field map estimation with by single‐point double‐shot. (2) Full pointwise encoding time reduction with radial acquisition. (3) Model‐based reconstruction with the $\Delta B_{0}$ obtained in step (1).

Our SPDS procedure also requires two acquisitions for field mapping, but it does not rely on echoes. Instead, each shot is a low‐resolution Cartesian acquisition (à la SPRITE^23^) where every point is acquired with the same encoding time (i.e., $T_{\text{d,}i}$,^1^ see panel 1 in Figure 3). These can be reconstructed either with ART or with an FFT. All κ‐space^2^ points in a given acquisition are subject to the same phase accumulation due to inhomogeneities. In this sense, each image has a unique phase in every voxel, and thus the phase difference between the two images is an exact proxy for the $B_{0}$ field. Note that the signal equation for a single shot is:

(2) $$s(\vec{\kappa })=\int d\vec{x}\;e^{-i\vec{\kappa }\cdot \vec{x}}e^{-i\gamma \Delta B_{0}(\vec{x})t(\vec{\kappa })}\rho (\vec{x}),$$

with $\Delta B_{0}(\vec{x})$ the deviation of the local field at position $\vec{x}$ with respect to $B_{0}$ (i.e., the inhomogeneity), and $t(\vec{\kappa })$ the time between spin excitation and the acquisition of signal at point $\vec{\kappa }$. Since all $t(\vec{\kappa })$ are equal for each acquisition, that is equivalent to $\rho_{i}$ (the low‐resolution ART reconstruction corresponding to shot i) having an extra global phase $-i\Delta B_{0}(\vec{x})T_{\text{d,}i}$, that is,

(3) $$s_{i}(\vec{\kappa })=\int d\vec{x}\;e^{-i\vec{\kappa }\cdot \vec{x}}{\tilde{\rho }}_{i}(\vec{x}),$$

with

(4) $${\tilde{\rho }}_{i}(\vec{x})=e^{-i\gamma \Delta B_{0}(\vec{x})T_{\text{d,}i}}\rho (\vec{x}).$$

The specific choice of a *global* $T_{\text{d,}i}$ for all signal acquisition points $\vec{\kappa }$ makes SPDS immune to geometric distortions due to $B_{0}$, and the field map can be obtained as

(5) $$\Delta B_{0}=\frac{\arg({\tilde{\rho }}_{2})-\arg({\tilde{\rho }}_{1})}{\gamma \left(T_{\text{d,2}}-T_{\text{d,1}}\right)}.$$

Notably, if these acquisitions are Cartesian and fully sampled, a discrete Fourier transform can be used. Furthermore, this method is compatible with arbitrary non‐Cartesian sequences, as long as $t(\vec{\kappa })$ is the same in each shot. Key to this discussion is that Equation (5) is an *exact* equality, while Equation (1) is an approximation which breaks down for strongly inhomogeneous fields.

After completion of this work, we found a sequence similar to SPDS published in a PhD thesis (mSPRITE^24^). The authors use mSPRITE to map $\Delta B_{0}$ distortions (up to 150 ppm) generated by metallic implants in an otherwise highly homogeneous $B_{0}$. To this end, they take a set of five κ‐space points by measuring at different $T_{\text{d,}i}$ within each readout gradient pulse, and produce five fully‐sampled SPRITE images. The off‐resonance map can then be obtained by fitting the phase evolution for each image pixel as a function of $T_{\text{d,}i}$. This is a single‐shot approach and therefore faster than SPDS. However, their κ‐space sampling and spacing differ among acquisitions, precluding simple Fourier analysis, affecting the resolution and FOV of the final field map, and thereby compromising its fidelity and the overall method performance.

For both GRE and SPDS, we use a masking procedure where we only keep $B_{0}$‐map points for which |ρ| is above a certain threshold. In this way we remove background noise. In our experiments these threshold values were adjusted individually for optimal results, but this can be automatized. After masking, each image is phase‐unwrapped with a MATLAB plugin.^25^ Finally, the pixel‐based $B_{0}$ map is fitted to a second‐ to fifth‐order polynomial, depending on the spread of the 95% confidence bounds of the coefficients and its performance when the polynomial function is used for ART‐based reconstruction. Note that low‐order fits lead to reduced Gibbs ringing in the resulting field maps.

The number of acquired κ‐space points ($\kappa_{\text{SPDS}}^{\max}$) affects the resolution of the final map, and has to be adjusted depending on the smoothness of the magnetic field inhomogeneities to be mapped, as well as on the length scale of relevant structures in the sample. We have found that 600 to 1200 points, reconstructed in a matrix size of 120 × 120 and FOV 6×6 cm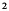 (resolutions in the range between 2.4 and 1.6 mm), suffice to resolve $\Delta B_{0}$ and faithfully mask the internal letters of the phantoms employed in this paper (2 mm thickness).

The procedure for field estimation by SPDS is depicted in Figure 3 (panel 1). The first SPDS maps were taken in the 260 mT scanner after intentionally spoiling the field by means of a single N48 grade NdFeB cubic magnet ($B_{\text{rem}}\approx 1.4$ T) of size (4 mm)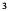, placed ≈1.5 cm away from the sample. This generates a field variation of ≈250μT within the circular phantom of diameter ≈40 mm. Before image acquisition, SPRITE sequences were executed consecutively with different dead times, $T_{\text{d,1}}=175\;\mu s$ and $T_{\text{d,2}}=250\;\mu s$, acquiring a set of 788 samples measured in the same κ‐space values and centered on a circumference of radius 250 m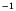. These datasets are reconstructed using ART with 10 iterations and λ=0.1 in a FOV of (6 cm)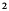 with a matrix size of 120×120. After that, the phase of the complex images is determined and masked, removing pixels with intensities <50% of the maximum absolute value of the image. We then unwrap the phase and use Equation (5) to estimate $\Delta B_{0}$ in the selected points. Finally, the data $\left\{{\vec{x}}_{i},\Delta B_{0}({\vec{x}}_{i})\right\}$ are fitted to a fifth degree polynomial. The time needed to reconstruct both datasets, phase unwrapping, masking, phase subtraction and fitting is <5 s in a standard CPU (Intel(R) Core(TM) i7‐6700 CPU @ 3.40 GHz). Note, however, that this depends strongly on the number of sampled points and can hence change for different FOV, phantoms structures, 3D acquisitions, etc. SPRITE acquisitions take approximately 40 s each.

#### 2.2.4 GRE versus SPDS

We also compared the field estimations by GRE and SPDS for varying degrees of inhomogeneity. Each scenario of inhomogeneity is generated by changing the configuration of N48‐grade NdFeB blocks in the 260 mT scanner, either adding more or simply bringing the magnets closer to the sample. To assess $\Delta B_{0}$ in each situation, GRE images of the hexagonal phantom (see Section 2.1) have been acquired in a FOV = (6 cm)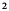 in a matrix size of N=60×60, with full κ‐space coverage and Fourier reconstruction. All GRE images have $T_{\text{acq}}=0.5$ ms and the lowest TE of each pair has TE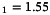 ms, approaching the maximum allowed by gradient coils and amplifier; meanwhile, TE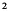 is chosen seeking a compromise between phase‐accumulation, which improves precision, with phase differences <2π between shots, which would otherwise invalidate Equation (5). We average longer for increasing inhomogeneities to compensate for SNR loss due to $T_{2}^{*}$ shortening: $N_{\text{avg}}=50$, 250, and 400 (scan times of 2.5, 12.5, and 20 min). SPRITE images have been acquired in a FOV = (6 cm)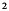 with a partial, low‐frequency coverage of κ‐space, reconstructed into a matrix size of N=60×60 with ART. Similarly to the GRE case, in SPDS we choose $T_{\text{d,1}}$ and $T_{\text{d,2}}$ and the number of acquired points according to the phase accumulation in each case, measuring $N_{\text{SP}}=235$, 521 and 492 points inside a circumference of radius 176, 214, and 210 m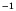. Here we also averaged depending on the inhomogeneity: $N_{\text{avg}}=9$, 16, and 50, for a total scan time of 2.8, 7, and 20 min for each shot, respectively.

### 2.3 Data acquisition and image reconstruction

#### 2.3.1 ART

Once a field map has been estimated, we fit $\Delta B_{0}$ to a polynomial function and run a full κ‐space PETRA acquisition, where the fitted function is used in the EM:

(6) $$E_{i,j}=\exp\left\{-i\left[{\vec{\kappa }}_{i}\cdot {\vec{x}}_{j}+\gamma \Delta B_{0}({\vec{x}}_{j})t({\vec{\kappa }}_{i})\right]\right\}.$$

The signal equation is thus s=Eρ. In our PETRA acquisitions, $t(\vec{\kappa })=T_{d}$ for the central point‐wise region, and $t(\vec{\kappa })=|\vec{\kappa }|/(\gamma G)$ for the radial part. For benchmarking purposes, we often reconstruct images omitting the PK, that is, we use the EM without the $\Delta B_{0}$ term. All reconstructions in this paper employ the Julia Programming Language,^26^ with CUDA.jl for GPU‐acceleration in an Nvidia GeForce GTX2080Ti card, with iterations $N_{\text{it}}=10$ and update parameter λ=0.1 (which takes ≈ 3 s). The only exception is the 3D acquisition in Figure 8 where, due to the computational time required, we set $N_{\text{it}}=\lambda =1$ (≈ 10 min).

#### 2.3.2 Simulations

We start by testing the performance of ART with PK coming from SPDS in fields with known distribution. To this end, we simulate the effect of running PETRA sequences on a two‐dimensional (2D) Shepp–Logan digital phantom subject to field distributions akin to those we use later in experiments. To prevent reconstruction crimes,^27^ the simulated signal takes the contribution of 10×10 spins per reconstruction pixel.

These simulations use Julia in the same GPU card as for reconstruction. Typical signal simulation times are ≈24 s, while one iteration in the ART reconstruction algorithm takes ≈13 s.

#### 2.3.3 2D projections

**FIGURE 4:**
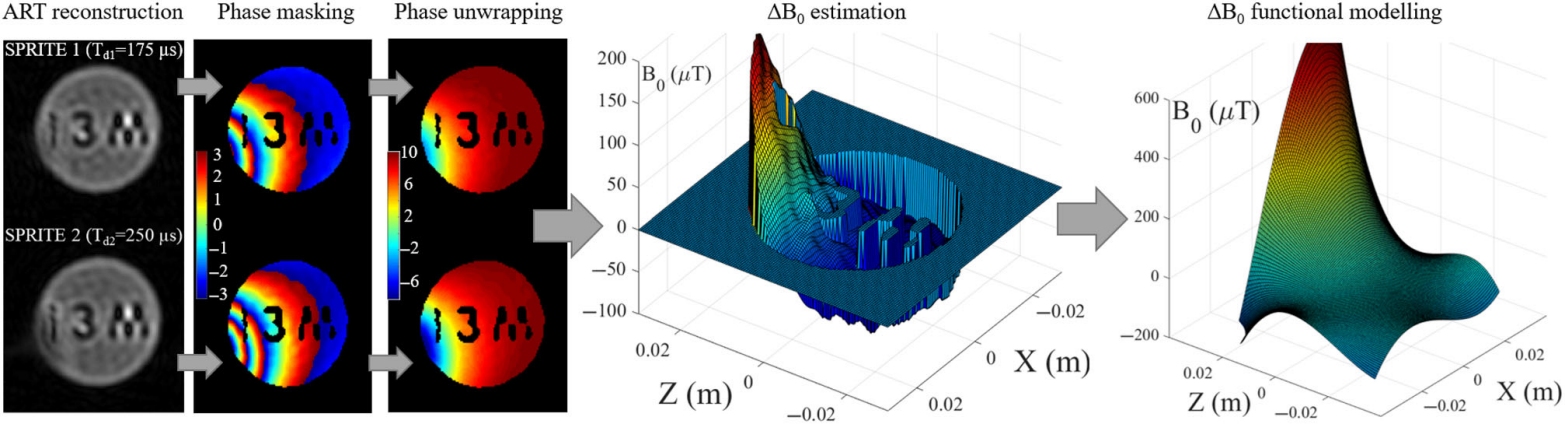
Steps for single‐point double‐shot mapping of the field inhomogeneity $\Delta B_{0}$. The first step is the acquisition and reconstruction of low‐resolution images with unique encoding times $T_{\text{d,1}}=175\;\mu s$ and $T_{\text{d,2}}=250\;\mu s$. Then, masking eliminates points from both images where signal intensity is below a given threshold with respect to maximum density of each image (50 % in this case). With the remaining points, we unwrap the phase and use Equation (5) to estimate $\Delta B_{0}$ at those points. Finally, we fit a fifth‐order polynomial to $\Delta B_{0}$.

For this first set of experiments, all PETRA and SPRITE images have been acquired in 2D. We consider different cases of $\Delta B_{0}$ inhomogeneity: (a) the 260 mT scanner (20 ppm inhomogeneity over a spherical region of 150 mm in diameter); (b) the 260 mT scanner, where we have superimposed a linear 15 mT/m gradient field along the z‐axis, that is, around half the value of the encoding gradient (≈29.4 mT/m); (c) the 260 mT scanner, where we place an NdFeB cube of N48 grade ($B_{\text{rem}}=1.4$ T) with volume (4 mm)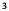, similarly to Figure 4; (d) the 197 mT scanner (roughly quadrupolar with curvatures ≈(0.2,−0.7,0.5) T/m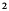), with the phantom along the xz and (e) yz planes; and (f) the 72 mT scanner.^9^ For each situation, we run a PETRA sequence and perform SPDS to obtain a $\Delta B_{0}$ estimation. Except for the 72 mT system, PETRA datasets were acquired in a FOV of 6×6 cm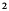 with full κ‐space radial coverage according to the Nyquist criterion and reconstructed into a matrix of 120×120 with ART. For these, we set $T_{d}=100\;\mu s$ and $T_{\text{acq}}=800\;\mu s$ for a maximum readout gradient strength of ≈29.4 mT/m. The PETRA dataset in the 72 mT scanner was acquired in a FOV of (20 cm)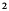 with full κ‐space radial coverage reconstructed into a matrix of 150×150 with ART. Due to longer coil ring‐down times and stronger heat dissipation at the gradients, we set $T_{d}=380\;\mu s$ and $T_{\text{acq}}=4$ ms for a maximum readout gradient strength of ≈2.2 mT/m. In all $\Delta B_{0}$ situations, we use a polynomial model to fit the pairs $\left\{{\vec{x}}_{i},\Delta B_{0}({\vec{x}}_{i})\right\}$ coming from SPDS, considering different orders and checking their performance when used for ART reconstruction with PK. We employed a fourth‐order polynomial for Figure 7A,C, third order for Figure 7F and second order for the rest, based on the perceived reconstruction quality. A possible extension would be to use orthogonal polynomials at fixed expansion order, or direct interpolation of the measured map.

#### 2.3.4 3D acquisition

We carried out also a model‐based ART reconstruction for a 3D acquisition in the 260 mT scanner. A first dataset was obtained after degrading $\Delta B_{0}$, again by close proximity of a N48 cube of NdFeB, and a second dataset was acquired in the homogeneous setting, after removing the NdFeB cube. Both datasets were fully sampled according to the Nyquist criterion, in a FOV of (6 cm)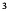 reconstructed into 120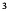 voxels, $T_{d}=100\;\mu s$, $T_{\text{acq}}=800\;\mu s$, $G_{\text{readout}}\approx 29.4$ mT/m, $N_{\text{radial}}=45,332$, $N_{\text{single}}=2,872$ (κ‐space samples for radial and single‐point readouts, respectively), TR=50 ms, $N_{\text{avg}}=9$ and $T_{\text{scan}}\approx 6$ h. Before removing the N48 magnet, we generate a 3D map of $\Delta B_{0}$ with SPDS, where the SPRITE sequences had $T_{\text{d,1}}=150\;\mu s$ and $T_{\text{d,2}}=200\;\mu s$, measuring a total of 8240 single points in a sphere of radius 210 m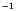 after five averages, for an overall scanning time of approximately 34 min. The resulting SPDS raw data was fitted to a fifth‐order polynomial.

#### 2.3.5 CP versus ART

For this comparison, we used the 260 mT scanner, with increasing linear inhomogeneity corresponding to intravoxel spectral bandwidths ${\text{BW}}_{\text{vox}}=0$, 106, 212, 426, 532, and 639 Hz/voxel (that corresponds to strengths of 0, 5, 10, 20, 25, 30 mT/m across 0.5 mm size voxels). We generate these inhomogeneities with intentional DC offsets on the currents through the z gradient coils. The PETRA acquisitions have common parameters: FOV = (6 cm)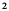, N=120×120, $T_{d}=100\;\mu s$, $T_{\text{acq}}=800\;\mu s$, BW=75 kHz, $G_{\text{readout}}\approx 29.4$ mT/m, $N_{\text{radial}}=376$, $N_{\text{single}}=232$, TR=50 ms, $N_{\text{avg}}=10$ and $T_{\text{scan}}=5$ min. The fixed 29.4 mT/m encoding gradient is equivalent to a spectral separation of 625 Hz/voxel. For completeness, we compare for each level of induced inhomogeneity (and therefore BW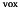) the reconstruction of the corresponding dataset using prior‐knowledge‐based ART against the well‐known Conjugate Phase method.^28^ To do so, we first interpolate the signal in κ‐space (also $t(\vec{\kappa })$) into a rectangular grid with ScatteredInterpolation.jl in Julia (see Section 3.2.4). After that, we follow the usual procedure, that is,

$$\rho ({\vec{x}}_{j})=\underset{i}{\sum }{\left(E_{i,j}\right)}^{*}s({\vec{\kappa }}_{i}),$$

with the $\Delta B_{0}$ inhomogeneity term in the conjugate phase's exponent, that is using Equation (6) for $E_{i,j}$.

#### 2.3.6 Robustness to noise

We study the performance of SPDS and reconstruction with PK against different levels of Gaussian noise in Figure 10A,B.

## 3 RESULTS

### 3.1 Field estimation

#### 3.1.1 Hall sensor

Mapping $\Delta B_{0}$ with a robot‐mounted Hall probe, as described in Section 2.2.1, proved to be experimentally challenging. Together with the need for a free parameter in the EM to relate the RF demodulation frequency (which we typically set to the center of the free induction decay spectrum) to the real Larmor frequency at the field center, all our attempts were unsatisfactory and only led to negative results.

#### 3.1.2 SPDS and GRE

Figure 4 shows the SPDS procedure for field mapping in 2D (Section 2.2.3), with $\Delta B_{0}\approx 300\;\mu T$ field variation.

**FIGURE 5:**
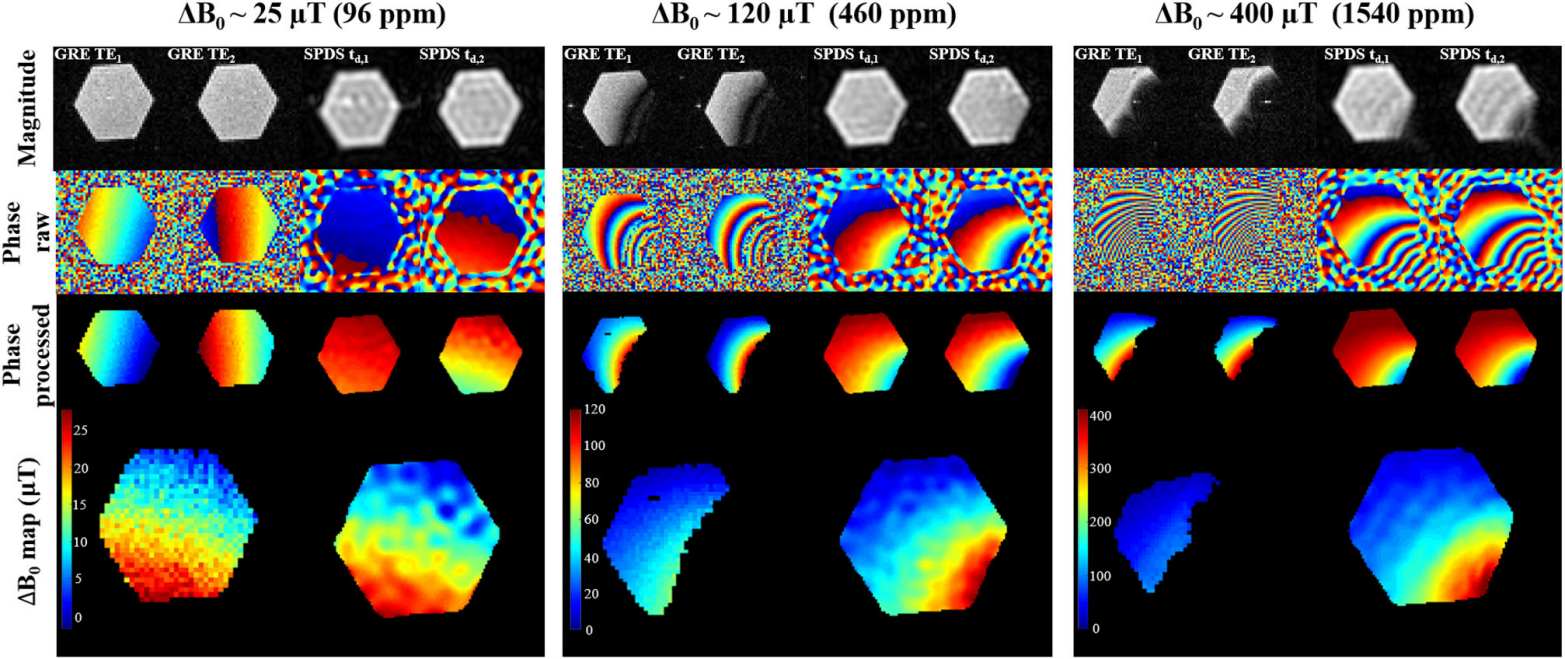
Comparison between single‐point double‐shot versus double‐shot gradient‐echo methods, for three different inhomogeneity levels and field arrangements. The upper row shows magnitude images corresponding to each shot. The second/third rows show phase images before/after masking and unwrapping. The lower row shows the estimated field inhomogeneity map.

In Figure 5, we compare the performance of SPDS against the classic approach of double‐shot GRE acquisition for different levels of induced inhomogeneity in the 260 mT scanner (Sections 2.2.2 and 2.2.4). We plot the absolute value of the two‐shot images, their wrapped and unwrapped phase and the estimated fields. Note that each column has a different configuration of extra NdFeB blocks that make $B_{0}$ intentionally inhomogeneous. We find SPDS more robust than GRE for middle (≈500 ppm) and high (≈1500 ppm) inhomogeneities.

### 3.2 Reconstruction with PK

#### 3.2.1 Simulations

**FIGURE 6:**
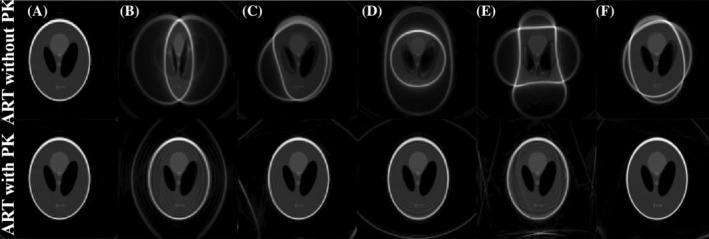
Performance of ART with perfect prior knowledge for field distributions explored experimentally (as in Figure 7), with a simulated Shepp–Logan signal with 100 spins per pixel.

Figure 6 shows reconstructions of a simulated Shepp‐Logan digital phantom with (bottom) and without (top) PK for field distributions similar to those used in the 2D experiments described in the next subsection. Artifacts are removed except for cases with rapid spatial field variations.

#### 3.2.2 2D projections

**FIGURE 7:**
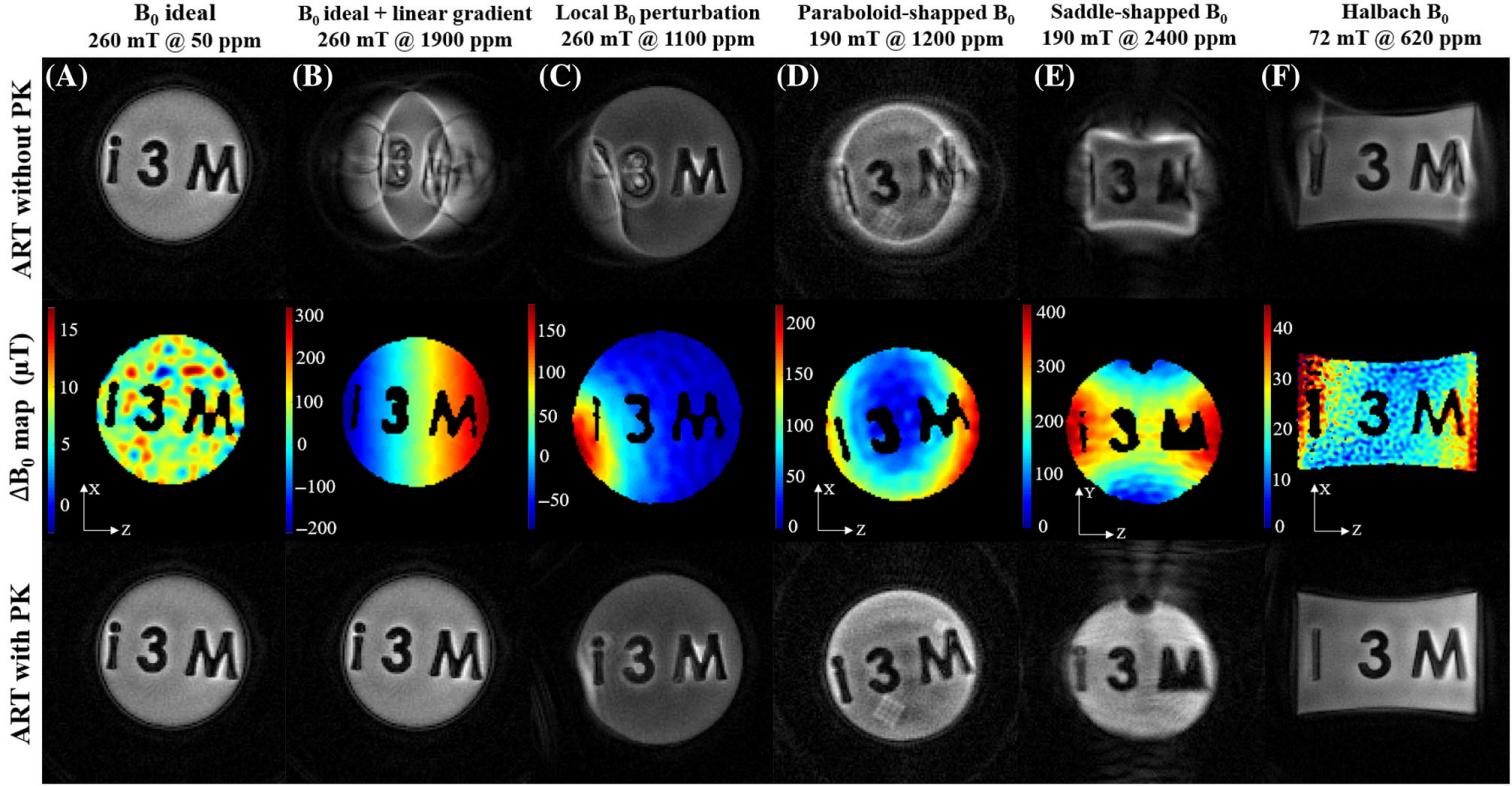
Performance of model‐based ART reconstruction (bottom row) when prior knowledge of the field inhomogeneity comes from single‐point double‐shot (middle row) for acquired two‐dimensional pointwise encoding time reduction with radial acquisition projections of a phantom in six different $\Delta B_{0}$ scenarios (see main text), as compared to reconstruction without prior knowledge (top row). There seems to be a small water infiltration inside phantom's polylactic acid (PLA) walls in (D), leading to a grid‐like structure. Also, in (E) we can see the inlet for liquids in the phantom, since it is placed in a vertical position.

Figure 7 shows that strong artifacts can be corrected by algebraic reconstruction when the SPDS field estimate is used as PK, in all three scanners and in situations where the field inhomogeneity is intentionally worsened by means of gradient fields or by bringing magnetic material close to the sample (see Section 2.3.3).

For each $\Delta B_{0}$ scenario, we show ART reconstructions without PK (top row), the resulting $\Delta B_{0}$ map from an SPDS acquisition (middle row) and ART reconstruction including PK (bottom row).

#### 3.2.3 3D acquisition

**FIGURE 8:**
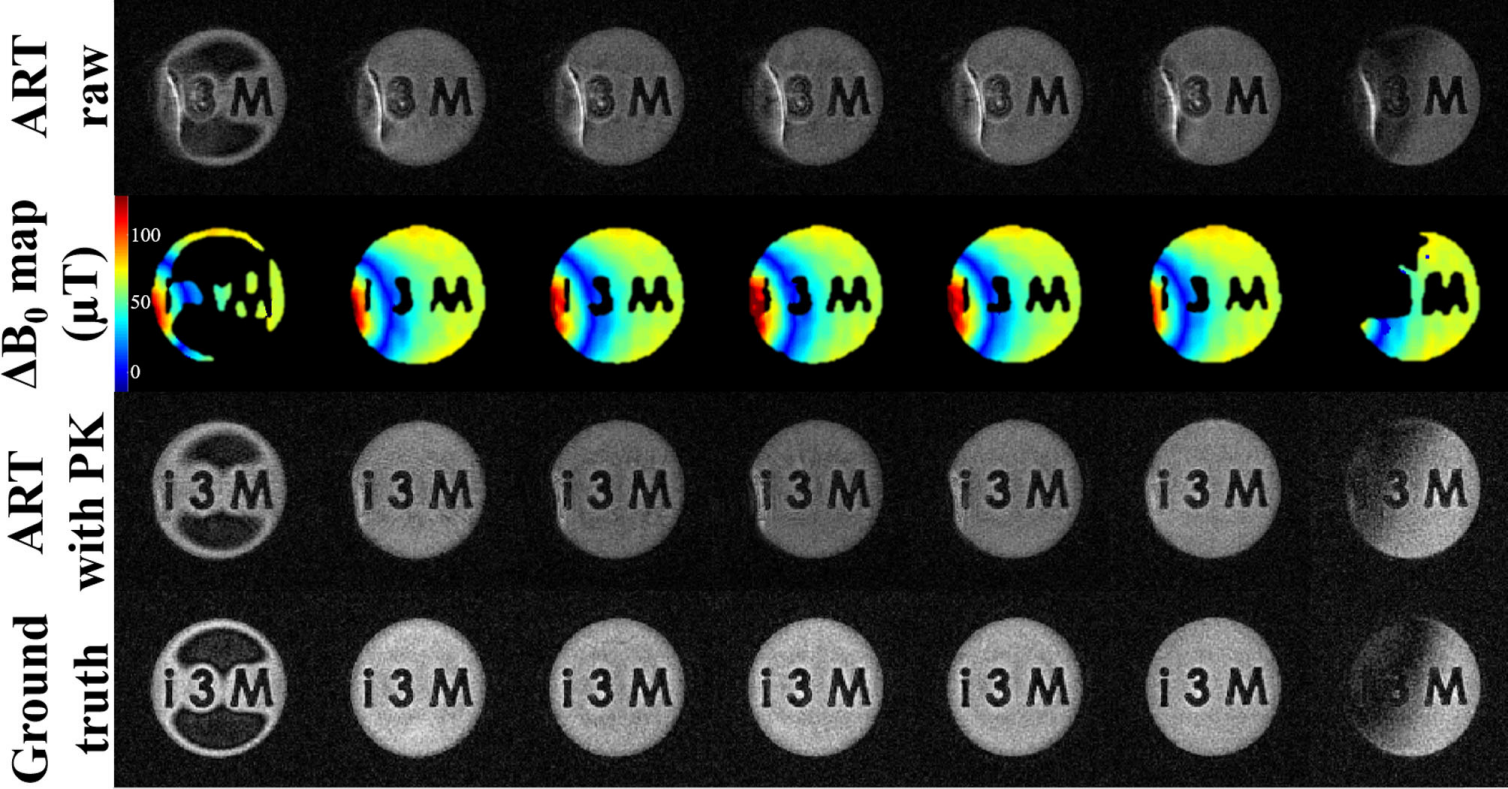
Perfomance of single‐point double‐shot (SPDS) for three‐dimensional pointwise encoding time reduction with radial acquisition in the 260 mT scanner with additional N48‐grade NdFeB magnet cubes to force a nonlinear field variation. In each column we show different y‐slices from the top of the phantom (where capillarity effects are notorious) to the phantom base. We show (first row) dataset 1 reconstructed with ART but without prior knowledge (PK) of $\Delta B_{0}$, (second row) $\Delta B_{0}$ estimation in each slice obtained with three‐dimensional SPDS mapping, (third row) dataset 1 reconstructed with ART adding PK of $\Delta B_{0}$ coming from a fifth‐order polynomial fitting of a SPDS map shown previously. After removing magnet cubes, we show (last row) dataset 2 reconstructed with ART without PK of $\Delta B_{0}$, which represents the ground truth due to the homogeneity of $B_{0}$. Each slice has brightness independently normalized to its maximum.

In Figure 8, we demonstrate the capabilities of SPDS mapping to create the PK of $\Delta B_{0}$ for a 3D PETRA acquisition (Section 2.3.4) with different y‐slices in each column of a phantom filled with 1% CuSO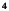 water solution in the 260 mT scanner. The ART reconstructions of dataset 1 with $\Delta B_{0}$ incorporated into the EM (third row) correct the large artifacts present in raw ART reconstructions without PK (first row) as compared to the ground truth (last row, where dataset 2 is reconstructed with ART without PK). A minor distortion is seen toward the left region, where homogeneity is most compromised.

#### 3.2.4 CP versus ART

**FIGURE 9:**
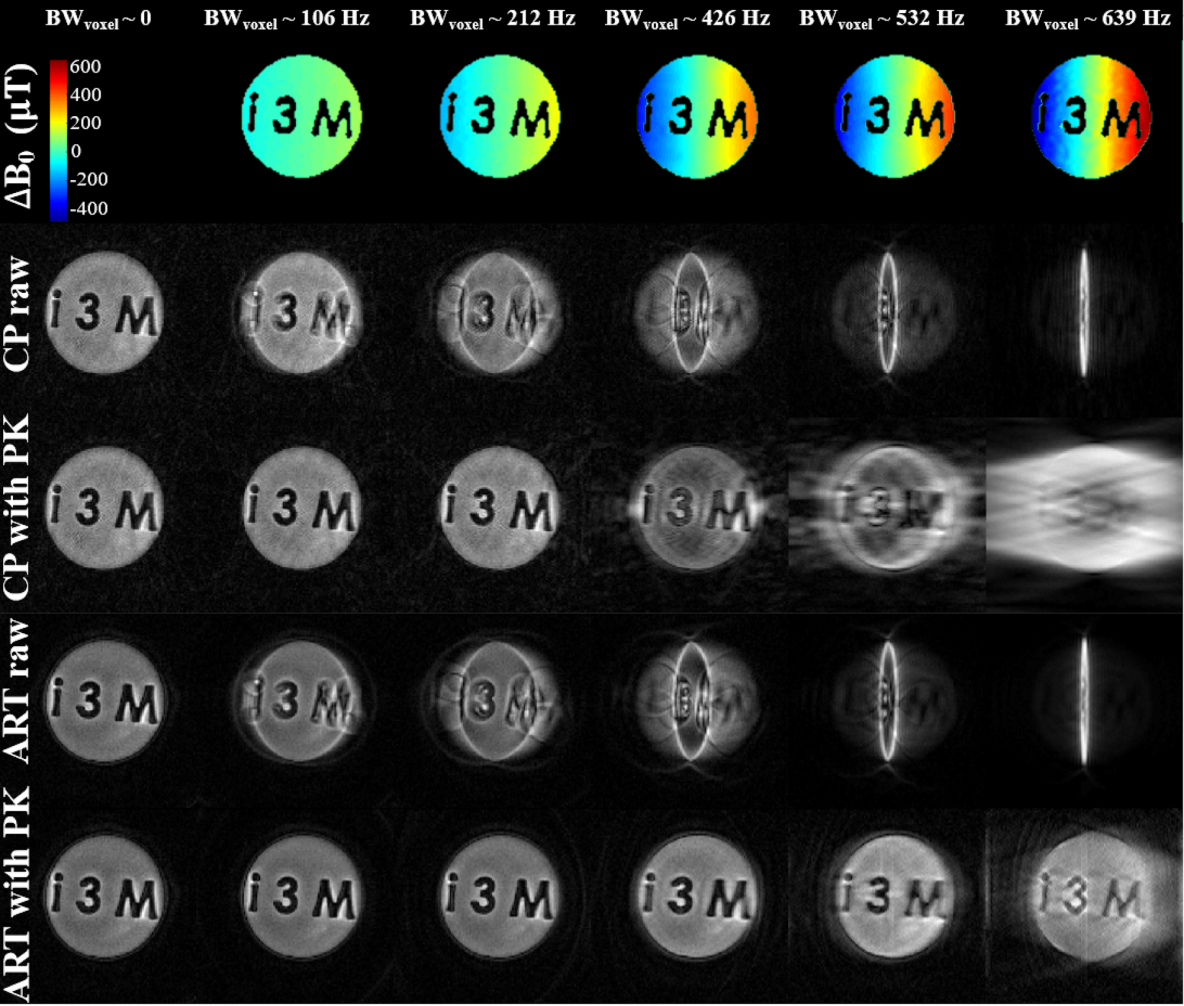
Experimental performance of ART and conjugate phase with increasing linear inhomogeneity, where single‐point double‐shot (SPDS) (field fitted to second order) is used to reconstruct pointwise encoding time reduction with radial acquisition datasets. All the images have been acquired with maximum gradient readout of ≈29.4 mT/m, for an intravoxel bandwidth of 625 Hz/voxel, while $\Delta B_{0}$ creates a frequency spread ranging from 0 to 639 Hz/voxel. First row: $\Delta B_{0}$ mapping obtained with SPDS. Second row: CP without prior knowledge (PK) reconstructions. Third row: CP provided of PK reconstructions. Fourth row: ART without PK reconstructions. Fifth row: ART with PK reconstructions.

Figure 9 shows CP and ART reconstructions in the presence of a linear inhomogeneity up to and beyond the strength of the ZTE encoding gradient (Section 2.3.5): ART is more robust to inhomogeneities than CP. All reconstructions have been performed with the SPDS estimate as PK for the EM and, since all linear inhomogeneities are well known, also with the exact analytical form $\Delta B_{0}(z)=g_{\text{inh.}}\cdot z$, finding basically no difference between them. Thus, Figure 9 is immune to uncertainties in the field estimate; however, in the results shown, the EM includes the field map obtained with SPDS.

#### 3.2.5 Robustness to noise

**FIGURE 10:**
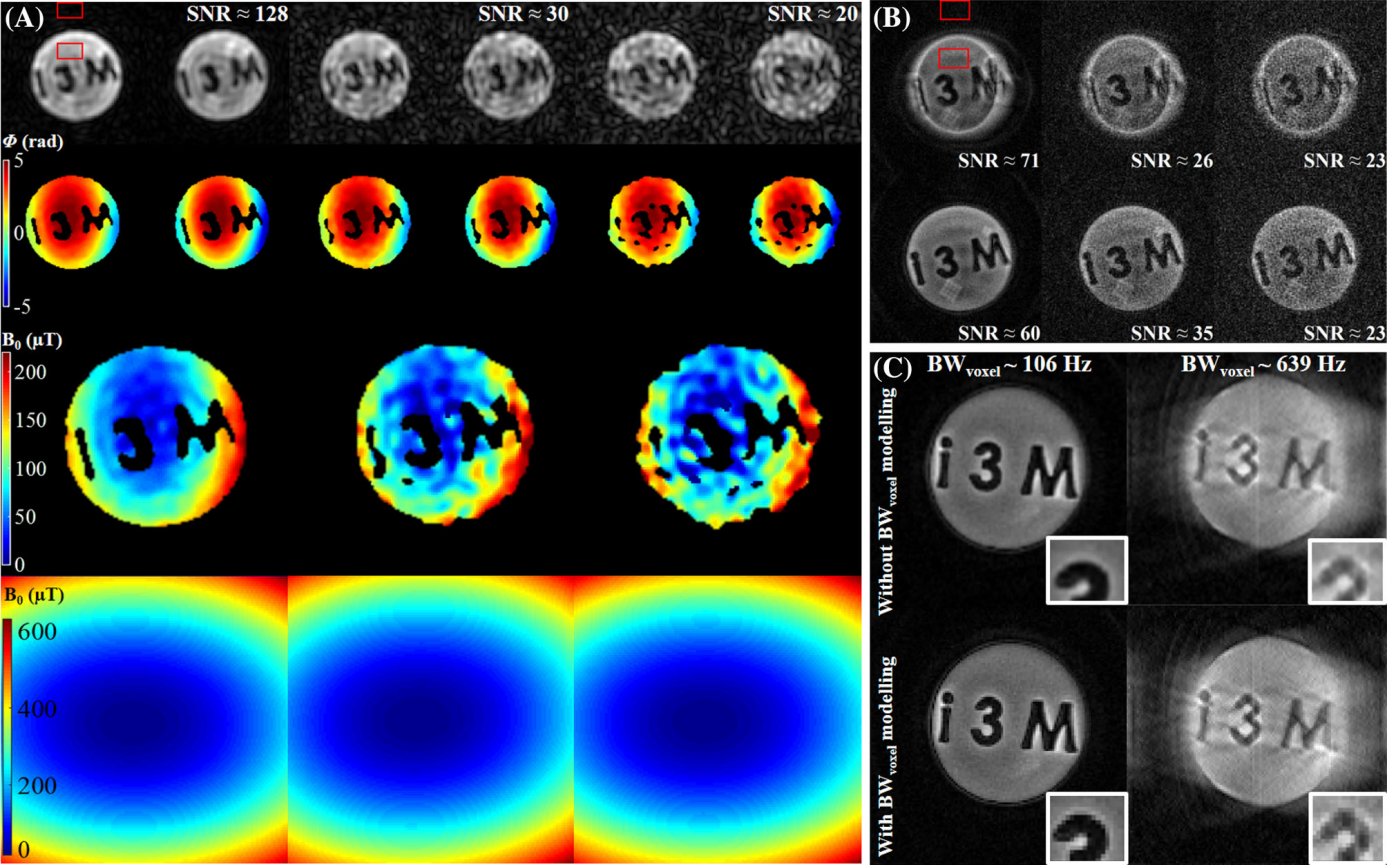
(A) Field estimation for the single‐point double‐shot (SPDS) acquisition in Figure 7D with Gaussian noise added to κ‐space, with red squares showing the two regions used to determine signal‐to‐noise ratio (SNR). First row shows magnitude images corresponding to each shot. Second row shows phase images after masking and phase unwrapping. Third row shows $\Delta B_{0}$ map obtained from Equation (5). Fourth row shows a second‐degree polynomial fit of $\Delta B_{0}$, where the coefficients are almost identical. (B) With the polynomial fit obtained from SPDS with SNR ≈128, we reconstruct with different levels of added Gaussian noise, without (upper row) and with (lower row) prior knowledge (PK). SNR level is determined from the two red squares. (C) Intravoxel field variation modeling can be included in the encoding matrix with simple position‐dependent sinc functions, leading to sharper edges. (Left) Linear inhomogeneity with BW
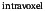
=106 Hz. (Right) Linear inhomogeneity with BW
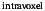
=639 Hz, as in Figure 9. Top/bottom: reconstruction without/with intravoxel field variation modeling. Insets show zoomed detail of the number 3, for better comparison.

Figure 10A shows that lowering SNR from 128 to 30 leads to worse field maps from SPDS. However, due to low‐order polynomial fitting, the resulting field is equal for all practical purposes.

## 4 DISCUSSION

### 4.1 Field estimation

The accuracy of field maps obtained with the THM1176 magnetometer is limited due to unknown tilts and oscillations of the rather long robotic arm, which is imposed by the scanner setup (see Figure 1 right). We tried to suppress these errors by adjusting the type of trajectory used to fill the volume, the delay between probe positioning and field measurement, and the velocity of the motor in each step. However, the mechanical reference between the probe and image space coordinates (determined by the gradient coils) is not precise enough for image reconstruction, unless the probe is attached to some element which can be visualized with MRI.

Additionally: (i) if the encoding gradient fields are also inhomogeneous, physical coordinates, and image‐space coordinates (given by gradients) will not coincide far away from gradient centers, leading to further uncertainties; (ii) the $\Delta B_{0}$ map is temperature dependent; (iii) the map has to be obtained with sufficient resolution, which can require many hours; and (iv) because the MRI signal is demodulated at an arbitrary frequency (typically the center frequency obtained by a simple free induction decay curve), the Hall probe map must be shifted by a constant term which needs to be found. While in principle all these difficulties can be overcome, we found this method impractical. All these effects make MRI‐based estimations of $B_{0}$ more reliable than direct field maps.

When comparing GRE with SPDS in Figure 5, we observe that GRE is not reliable close to the source of high inhomogeneity, due to the large intravoxel $T_{2}^{*}$, and the image phase is not smooth in that region. Instead, SPDS works even for ≈1,500 ppm (400μT in 260 mT). SPDS is limited by the time required to switch from trasmit to receive mode in the RF chain (dead‐time), since $T_{2}^{*}$ leads to a drop in SNR in the meantime. On the other hand, GRE includes the additional duration of half the acquisition window, phase encoding and the gradient switching time. While SPDS requires the encoding gradient to be on before spin excitation (à la ZTE), GRE is based on gradient switching after RF excitation, and thus it is harder to shorten phase and encoding times due to inaccuracies given by, for example, eddy currents, leading to further hardware constraints. This highlights that, in situations of large inhomogeneities, the GRE method can be SNR limited. In addition, SPDS does not suffer from geometric distortions in the field map, also favoring SPDS in medium‐inhomogeneity regimes.

We also produced field maps with reduced κ‐space sampling for all three configurations in Figure 5. To this end, we acquired only $N_{\text{SP}}=120$ κ‐space points (as compared to $N_{\text{SP}}=235$, 521, and 492). This led to compatible results, except for $\Delta B_{0}\approx 400\;\mu T$, where the lower spatial resolution complicated masking around the bottom‐right corner. The minimum number of κ‐space samples is therefore determined by SNR level, masking and phase unwrapping stages, even if field inhomogeneities can be described by smooth (low‐order) polynomials. The relevance of a reliable $\Delta B_{0}$ map is apparent from the comparison between Figures 6 and 7, especially at borders or regions with abrupt field changes. A better map can be obtained either by increasing SPDS resolution (i.e., $\kappa_{\max}^{\text{SPDS}}$), or by mapping a larger FOV with a bigger phantom. Another potential solution would be an iterative procedure, where the field estimate is updated at each step by consistency with the double‐shot signals.^29^, ^30^ Still, one advantage of our method is that it does not require iterative methods.

On the other hand, SPDS follows a pointwise scheme and is therefore inherently slower than GRE. Fortunately, $B_{0}$ distributions tend to be smooth and sampling small numbers of κ‐space points usually suffices. This can lead to Gibbs ringing in borders, but low‐order polynomial fits suppress such oscillations. Furthermore, it also makes it resilient to noisier acquisitions (see Figure 10A).

To minimize the acquisition time needed to perform SPDS, it is important to find a balance between the number of averages and the points sampled. The former helps to improve the SNR of the image, which is crucial to get reliable pairs $\left\{{\vec{x}}_{i},\Delta B_{0}({\vec{x}}_{i})\right\}$, whereas the latter provides higher nominal resolution, facilitating the masking step in structured phantoms and enabling finer spatial details for cases where $\Delta B_{0}$ has abrupt variations. In Figure 4, each SPRITE sequence lasts 39 s for a total time of around 80 s for the SPDS protocol. This could be reduced using shorter repetition times and Ernst angle excitation.

There is also a compromise when choosing the difference $T_{\text{d,2}}-T_{\text{d,1}}$. Ideally, this difference should be long in order to maximize the accuracy of $\Delta B_{0}$, but the relative phase between shots cannot exceed 2π, as the obtained field would otherwise have an unknown scaling factor, as per Equation (5). Furthermore, as can be seen in Figure 4 (right), the polynomial fits typically diverge outside of the fitted region. Thus, a calibration of the largest possible imaging region with a dedicated homogeneous phantom, with a sequence calibrated for maximum phase difference, is preferable.

In terms of total acquisition time for field estimation, the complete 3D SPDS protocol for Figure 8 took around 68 min, plus several minutes for data treatment as previously explained (Figure 4). While this may be excessively long for many applications, it is probably the only reasonable option in regimes of such extreme inhomogeneity. Note that this is still shorter than direct field mapping with a Hall probe, and a single calibration can be valid for a long time, as long as there are no significant field changes. Shorter acquisitions should be possible with hybrid^31^ or compressed sensing^32^ schemes.

### 4.2 Image reconstruction with PK

In all cases in Figure 7 we see a major improvement as compared to reconstruction with no PK of the field, except for cases where the field changes abruptly such as (C), where we see a small distortion at the bottom‐left border, or (F) also near the borders (the image is non‐rectangular due to nonlinear gradients, but this could also be incorporated into the EM). The strongest artifacts are present in (E), in addition to a small tilt of the sample which leads to a nonideal reconstruction even with SPDS. However, note that in (B) the total field variation across the phantom is ≈500μT and reconstruction is close to ideal, while in (E) a variation of ≈400μs introduces significant artifacts. Two possible reasons for this are: (i) the SPDS estimate is not good enough or (ii) there is a hard physical limit for reconstructability even with perfect field knowledge. Simulations in Figure 6 show no shape distortion in the outer ellipse of the phantoms, and thus provide no explanation to the small deformation in the lower left part in Figure 7C. This means that SPDS is partially failing when the field has abrupt spatial changes. On the other hand, in Figure 6C,E we also observe artifacts within the phantom, precisely where the field changes most abruptly.

At a given level of inhomogeneity, the encoding gradient is below the intravoxel bandwidth, and thus reconstruction is not possible even with perfect field knowledge. On the contrary, with small intravoxel bandwidth and large intervoxel bandwidth (an unrealistic scenario), perfect knowledge of the field would result in artifact‐free reconstruction.

Large intravoxel bandwidths (short T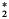) lead to blurring. Incorporating the known intravoxel field variation (to first order) leads to an EM

$$E(\vec{\kappa },{\vec{x}}_{i})=\text{sinc}(\frac{{\tilde{\kappa }}_{x}dx}{2})\text{sinc}(\frac{{\tilde{\kappa }}_{y}dy}{2})e^{-i\vec{\kappa }\cdot {\vec{x}}_{i}-i\frac{\left|\vec{\kappa }\right|}{G}\Delta B_{0}({\vec{x}}_{i})}$$

with

$${\tilde{\kappa }}_{x}=\kappa_{x}+\frac{|\vec{\kappa }|g_{\text{inh.}}}{G}$$

$${\tilde{\kappa }}_{y}=\kappa_{y}$$

in the case of linear inhomogeneity (see Figure 9), and results in sharper edges (see Figure 10C). We have not included this modeling since it improves slightly reconstruction quality but does not solve artifacts. Note also that $T_{2}$ decay could be incorporated, but it is negligible for our acquisition windows.

Finally, adding noise does not disturb the capability of SPDS+ART to remove artifacts, as can be seen in Figure 10B.

### 4.3 Limits of reconstruction

We are not aware of previous comparisons between model‐based reconstructions and Conjugate Phase reconstructions (CP,^28^ often used in non‐Cartesian MRI) in the presence of strong field inhomogeneities.

Comparing the CP and ART reconstructions in Figure 9 we observe that: (i) conjugate phase fails sooner than ART, roughly when the inhomogeneity is half the value of the encoding gradient, while ART is able to reconstruct up to the level of the encoding gradient, even if it does so with severe artifacts and (ii) perfect PK does not prevent massive artifacts when the local inhomogeneity is at the encoding gradient strength.

### 4.4 Outlook

SPDS field estimation is based on point‐wise encoding and can take longer than a standard double‐shot GRE, but it might be the only option for special‐purpose hardware. In this case, iterative algorithms can be used to obtain a nondistorted field map,^29^, ^30^ while SNR drops need to be recovered by expensive increases of signal averaging. In addition, we have shown that low‐resolution $\Delta B_{0}$ field maps usually suffice, and the problems encountered with our method had to do with phantom edge effects and the masking/unwrapping steps. The method could be used for sporadic calibrations of the field, for example, with an homogeneous sample fills the full region of interest, so multiple acquisitions with the same $B_{0}$ map can follow, as long as the field is stable and local permeability effects in the sample can be disregarded.

This work stemmed from the need to reconstruct images from a highly inhomogeneous scanner designed for in vivo dental imaging, but its impact can be extrapolated to most LF‐MRI systems, where $B_{0}$ tends to be less homogeneous than in high‐field scanners and related artifacts are usual.^30^ In non‐Cartesian sequences inhomogeneities lead to blurring, brightness concentration and other deleterious effects. Furthermore, even more disruptive designs, such as gradient‐free scanners^33^ or hand‐held devices,^34^, ^35^ though not amenable to SPDS field estimation, seem to lead to an increasing level of experimental imperfections that demand advanced reconstruction algorithms.^36^ For all these reasons, it is desirable to find robust methods to cope with strong field inhomogeneities and non‐Cartesian sequences.

## 5 CONCLUSIONS

We have introduced SPDS, a magnetic‐field mapping method based on double shot SPRITE sequences. Compared to double shot GRE, SPDS does not suffer from incorrect coordinate assignment and it is more robust against eddy currents and SNR drops, lending itself to situations where the main magnetic field is largely imperfect. We have shown that this method provides reasonable field estimations in situations with inhomogeneities as large as 1000 ppm over circular samples of only 2 cm radius (Figure 7D). We have also shown that model‐based reconstruction by ART (Kaczmarz's algorithm), with the SPDS field estimate as prior, is able to remove artifacts up to levels approaching the regime where the encoding gradient is no longer able to spectrally resolve the set of Larmor frequencies of spins in two adjacent voxels. We have approached this limit both with linear and nonlinear inhomogeneities, and found that almost up to 2000 ppm nonlinear inhomogeneity in a phantom filling a FOV of (6 cm)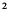 can be corrected (Figure 6E). For linear inhomogeneities, we have seen that one can reconstruct a recognizable image *beyond* this point, even if some artifacts are apparent. On the contrary, Conjugate Phase reconstruction and Fourier methods fail.

## FUNDING INFORMATION

This work was supported by the Ministerio de Ciencia e Innovación of Spain through research grant PID2022‐142719OB‐C22 and the European Innovation Council (EIC‐Transition 101136407). Action co‐financed by the Agencia Valenciana de la Innovación (INNVA1/2022/4 and INNVA1/2023/30). JB acknowledges support from the Innodocto program of the Agencia Valenciana de la Innovación (INNTA3/2021/17).

## CONFLICT OF INTEREST STATEMENT

JB was a researcher at Tesoro Imaging S.L. during a part of this work.

## ENDNOTES

1 We import the term “dead time” $T_{d}$ from ZTE for SPDS, as it is also determined by electronic ring‐down and blanking effects.

2 In this work, we intentionally use the notation κ‐space, rather than k‐space, to highlight the fact that off‐resonant effects are extreme and preclude most well‐known k‐space properties, such as the fact that resolution is constant across an image and is determined by $k_{\max}$.
